# Teeth discolouration and prescribed slimming magistral formula: a case report

**DOI:** 10.1080/07853890.2021.1897390

**Published:** 2021-09-28

**Authors:** Tânia Fernandes, Armanda Amorim, Ana I. Fernandes

**Affiliations:** a PharmSci Lab, CiiEM - Centro de Investigação Interdisciplinar Egas Moniz (CiiEM), Egas Moniz Cooperativa de Ensino Superior, Caparica, Portugal; b Centro de Investigação Interdisciplinar Egas Moniz (CiiEM), Egas Moniz Cooperativa de Ensino Superior, Caparica, Portugal

## Abstract

**Introduction:**

Tooth discolouration is a common subject of aesthetic dissatisfaction for which patients seek dental care [1,2]. Discolouration can be extrinsic (chromogenic agents deposited on the tooth surface – enamel), intrinsic (chromogens deposited within the bulk of the tooth – dentin), and internalised discolouration, a combination of both [1,2]. Different factors can be accountable for dental staining, such as certain medicines, smoking, some foods/beverages (e.g. coffee, tea, wine), poor oral hygiene (e.g. chromogenic bacteria), advancing age, trauma or disease [3]. Medicines known to discolour teeth, especially during tooth development, include antibiotics, antihistamines, antihypertensives, antipsychotics and fluoride [2,3]. Yet, there are numerous drugs with scarce information in literature about its potential for tooth discolouration. To the best of our knowledge this is the first report of a patient´s tooth discolouration by staining, suspected to result from a prescribed magistral formula (MF) for weight loss.

**Materials and methods:**

This study details a case of a non-smoking 51-year-old Caucasian woman presenting severely stained teeth and a clinical history of depression and pre-obesity, medicated with escitalopram (20 mg; antidepressant) and loflazepate (2 mg in SOS; anxiolytic). The patient referred taking hard gelatine capsules for weight loss (once daily for 3 weeks), prescribed as a MF; each unit was composed of chlordiazepoxide (8 mg; anxiolytic), phenolphthalein (PhP; 65 mg; laxative), furosemide (20 mg; diuretic), metformine (280 mg; antidiabetic), bupropion (120 mg; antidepressant), artichoke extract (110 mg; allegedly choleretic and diuretic), *Citrus aurantium* extract (150 mg; claimed stimulant and thermogenic). Written consent for data use was obtained from the patient.

**Results:**

Several teeth presented discolouration by staining as dark brown spots, which together with the clinical history, allowed the establishment of aetiology and selection of treatment. The dental cleaning procedure (with ultrasound and final polishing with a zirconium silicate particles-based prophylaxis paste, without fluoride) was effective. Slimming MF was discontinued and staining did not reappear (up to 3 months), even though the patient maintained her other habits. Neither drugs, nor botanicals, in the patient’s MF were identified in literature as tooth stain-causing molecules.

**Discussion and conclusions:**

The colour and type of staining is consistent with deposition of chromogens in tooth crests. Xerostomia, a common side-effect of the antidepressants, may have potentiated the chromogens' deposition, although the medicine did not enter in close contact with teeth. A systemic manifestation of the drug, as a result of a combination of intrinsic and extrinsic factors, is also possible. Stains may have originated from tainted plant extracts (containing tannins or uncontrolled/unlabeled heavy metals − Fe, Cu, Cd, Sn − due to phytogeography; contaminants) or PhP (an acid–base titration reagent, changing colour according to pH), here used as a stimulant laxative, withdrawn from medicines in many European countries due to its carcinogenic potential. The hypotheses raised warrant further investigation and clarification of the role of drugs/botanicals and contaminants in tooth staining.
